# Review of Satralizumab for Neuromyelitis Optica Spectrum Disorder: A New Biologic Agent Targeting the Interleukin-6 Receptor

**DOI:** 10.7759/cureus.55100

**Published:** 2024-02-27

**Authors:** Bikash R Meher, Rashmi R Mohanty, Ashish Dash

**Affiliations:** 1 Pharmacology, All India Institute of Medical Sciences, Bhubaneswar, IND; 2 General Medicine, All India Institute of Medical Sciences, Bhubaneswar, IND

**Keywords:** neuromyelitis optica spectrum disorder, nmosd, il-6 antagonist, monoclonal antibodies, satralizumab

## Abstract

Currently, three monoclonal antibodies (MABs) have received regulatory approval from the federal agency, the United States Food and Drug Administration (USFDA), for the medical management of neuromyelitis optica spectrum disorder (NMOSD). Satralizumab was the third approved therapy after MABs like eculizumab and inebilizumab for NMOSD, an uncommon but severe enfeebling autoimmune neurological disease. Satralizumab, a humanized monoclonal antibody, exerts its action in NMOSD by acting against cytokine interleukin-6 (IL-6), a foremost mediator in the pathological process of NMOSD. Two pivotal clinical trials carried out in NMOSD patients had established that satralizumab significantly decreased the rate of relapse in patients suffering from NMOSD as opposed to placebo. The trials also demonstrated that satralizumab is relatively safe. Thus, satralizumab provides an efficacious and safe treatment option for this rare, disabling central nervous system (CNS) disease. Our review aimed to elucidate the pharmacological characteristics of satralizumab and illustrate the available evidence regarding its safety and efficacy in patients with NMOSD.

## Introduction and background

Neuromyelitis optica spectrum disorder (NMOSD) is an uncommon but serious autoimmune disorder [1,2]. Optic nerves and the spinal cord are primarily affected by NMOSD; however, it can also involve other brain regions like the thalamus and dorsal medulla [3,4]. Recurrent inflammation of the brain and spinal cord are common manifestations of NMOSD, causing progressive loss of vision and motor disability [5-7]. Uncontrollable vomiting, intractable hiccups, excessive daytime sleepiness, and seizures in children are a few other noticeable symptoms associated with this rare disease [8-10]. Approximately 70% of NMOSD patients experienced relapse within two years of disease without effective preventive therapy, and it is a matter of grave concern as it causes irreversible neurological damage [11]. Epidemiological data suggest that the prevalence of NMOSD ranges from 0.5 to 4 per 100,000 and has a high propensity for the female gender [12]. The median onset age is 40 years; however, in any age group, patients can be affected by this disorder, including children, young adults, or adults over 65 years [13]. As far as ethnicity is concerned, NMOSD is more frequent in the African and Asian populations than in the White populations [14]. Approximately 60% to 80% of affected patients develop autoantibody against aquaporin-4-IgG (AQP4-IgG) [15]. The pathophysiology of NMOSD is complex, and various factors have been implicated in it. In addition to the association of human leukocyte antigens (*HLA*) and aquaporin-4 (*AQP4*) genes, many other genes have also been implicated in the etiopathogenesis of NMOSDs.

Furthermore, various other factors, such as dietary habits and life, have an association with the onset of this disease [16]. Emerging evidence suggests that cytokine-like interleukin-6 (IL-6) is a pivotal player in the pathogenesis of this CNS disorder [17]. IL-6 is a pleomorphic pro-inflammatory cytokine produced by different cells like B and T cells, fibroblasts, monocytes, and mesangial cells.

It plays an important role in acute inflammation, host defense mechanisms, and immune responses [18]. IL-6 triggers the signaling cascade by forming a glycoprotein 130 (gp130) complex through its receptors, membrane-bound IL-6R, and soluble IL-R [19]. IL- 6 may promote the pathogenesis in NMOSD by various processes such as activation and differentiation of naive T cells into pro-inflammatory type helper T cells, differentiation of B cells into AQP4-IgG producing plasmablasts, and production of pathogenic AQP4-IgG. These lead to inflammation, impairment of blood-brain barrier function, and damage to astrocytes [20].

**Figure 1:**
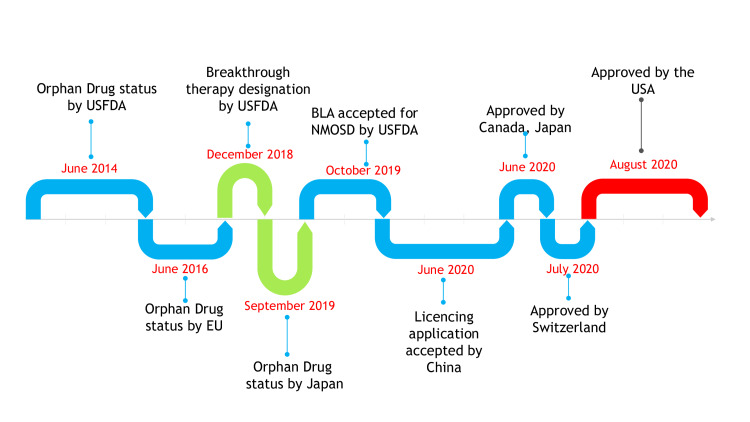
Timeline of satralizumab USFDA: United States Food and Drug Administration; EU: European Union; NMOSD: Neuromyelitis optica spectrum disorder; BLA: biologics license application Image courtesy: Dash A

The goal of drug therapy in NMOSD is to manage the acute episode and prevent future relapse so that long-term disability and sequelae can be checked [21]. Until recently, no specifically approved drug was available to treat NMOSD, and their management by different immunosuppressive drugs like mycophenolate mofetil (MMF) and azathioprine (AZA), or mouse-human chimeric monoclonal antibody (MAB) like rituximab (RTX) was off-label [22]. However, two-thirds of patients treated with MMF and AZA experience relapse, emphasizing the need for additional effective drugs [6]. Of late, many new biological agents have been evaluated for the management of NMSOD based on the pathogenesis of NMSOD. Eculizumab monoclonal antibody targeting C5 complement protein was the first therapy approved by the USFDA for NMOSD in AQP4 antibody-positive adult patients [23]. Inebilizumab, which is a humanized monoclonal targeting B cell-specific antigen CD19, was the second drug to be approved therapy by the USFDA to use in adult patients with NMOSD who are found to be anti-aquaporin-4 or AQP4 antibody positive) [24]. Satralizumab was approved in 2020 and was the third monoclonal antibody after eculizumab and inebilizumab to receive the nod from the USFDA for treating NMOSD [25]. It has also been approved in Japan for adults and children suffering from AQP4 antibody-positive NMOSD for the prevention of recurrence and relapse [26]. Similarly, it has also been approved in countries like Canada and Switzerland for managing patients suffering from NMOSD [27-29]. The timeline of satralizumab drug development is illustrated in Figure 1.

In this review, we have summarised the pharmacological properties of Satralizumab and its efficacy and safety in patients with NMOSD.

## Review

Data sources, extraction, and selection

We searched different databases like PubMed and Cochrane Library for relevant articles on satralizumab in NMOSD from inception till December 2023 using keywords like "Satralizumab" [All Fields] AND ("Neuromyelitis optica spectrum disorder" [All Fields] OR "NMOSD" [All Fields]). We also reviewed product information and checked the International Clinical Trials Registry Platform (ICTRP) for unpublished data. For this review, we focused on the studies evaluating the efficacy and safety of satralizumab in NMOSD patients. Furthermore, we also included literature related to this drug's chemistry, pharmacokinetics, and pharmacodynamics properties.

Chemistry

Satralizumab is a humanized IgG2 monoclonal antibody consisting of two heavy chains (443 amino acid residues each) and two light chains (214 amino acid residues each). It is a glycoprotein produced through recombinant DNA technology in mammalian cell lines like Chinese hamster ovary cells. Its molecular formula is C6340-H9776-N1684-O2022-S46, and its molecular weight is 143 kDa [30]. It is designed by an innovative process, "antibody recycling technology", which enables it to have a longer duration of action. The other names of satralizumab are SA-237, RG 6168, and satralizumab-mwge. It is developed by Chugai Pharmaceutical and Roche and marketed under the brand name "Enspryng" for treating NMOSD. It is available as 120 mg/ml in a single-dose prefilled syringe [31].

Pharmacokinetics

The pharmacokinetic properties of satralizumab were assessed in healthy volunteers and SD patients of varying ethnic groups. The population pharmacokinetic (pop PK) analysis method was developed using data from 154 patients and used to predict individual PK parameters [30-32].

The bioavailability of satralizumab following 120 mg subcutaneous injection was 85%. The maximum concentration (Cmax) was 31.5 mcg/ml, and the area under the curve (AUC) at steady state was 737 mcg.ml/day, respectively, and steady-state was achieved after eight weeks of the loading period. It had biphasic distribution, and the volume of distribution was estimated at 3.46 L and 2.07 L for central and peripheral compartments, respectively. It exhibited concentration-dependent clearance (0.0601L/day), and its terminal half-life was about 30 days with a range of 22- 37 days.

As monoclonal antibodies are mostly cleared by catabolism, a metabolism study was not conducted directly for satralizumab. Studies were also not conducted to see whether the pharmacokinetics of satralizumab were affected by renal and hepatic dysfunction. Drug-drug interaction studies were also not carried out to observe the potential interaction between satralizumab and any concomitant drugs. The pharmacokinetic study suggested that factors like race, gender, and age did not influence the PK properties of the drugs. However, body weight seemed to have some influence [30-32]. The PK properties of the drug are summarised (Table 1).

**Table 1:** Pharmacokinetic of satralizuamb AUC: Area under the curve

Absorption	Distribution	Metabolism	Elimination
Bio-availability: 85%; Cmax: 31.5 mcg/ml; AUC: 737 mcg.mL/day	Biphasic distribution volume of distribution: Central: 3.46L Peripheral: 2.07L	It has not been studied directly, as antibodies are cleared mainly by catabolism	Mean terminal half-life: 30 days (22-27 days)

Pharmacodynamics

**Figure 2:**
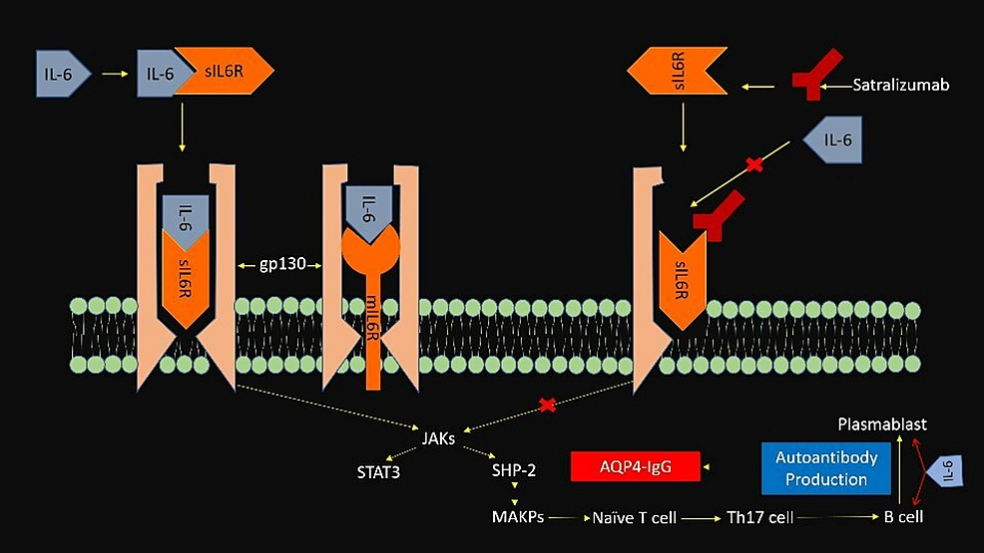
Mechanism of action of satralizumab Abbreviations: AQP4-IgG- Aquaporin-IgG; sIL6R-Soluble interleukin 6 receptor; mIL6R- Membrane-bound interleukin 6 receptor; JAKs- Janus Kinase; STAT- Signal transducer and activator of transcription; MAPS-Mitogen activated protein kinase; SIP-2- SymRK interacting protein- 2 Picture courtesy: Dash A

IL-6 binds to both soluble and transmembrane IL-6 receptors and initiates the IL-6-mediated signaling system. Satralizumab exerts its action by binding to both soluble and transmembrane IL-6 receptors on the surface of plasmablasts and blocks the binding of IL-6 to both the receptors, thereby inhibiting IL-6 mediated signal transmission and, consequently, the downstream pathway implicated in the pathogenesis of NMOSD (Figure 2) [29,33].

Therapeutic efficacy

The efficacy of satralizumab in managing patients suffering from NMOSD was demonstrated by two trials: SAkuraSky and SAkuraStar. Both trials by design are multicentre phase 3, randomized, parallel design, double-blind, placebo-controlled study followed by an open-label extension period [34,35]. SAkuraSky trial was conducted early, where satralizumab was assessed as an add-on therapy to immunosuppressive agents, whereas the SAkuraStar trial was conducted later and compared the satralizumab monotherapy with a placebo [34,35]. In both studies patients, neuromyelitis optica patients, as defined by 2006 Wingerchuk criteria, regardless of AQP4-IgG expression or AQP4-IgG seropositive NMOSD patients with either single or recurrent events of myelitis or optic neuritis, were included [36,37]. Furthermore, to be eligible to participate in the study, patients should have had at least one episode of relapse in the preceding one year and have a score of 0 to 6.5 on the Expanded Disability Status Scale (EDSS) at the time of screening. Patients who have received therapy targeting the interleukin-6 pathway were precluded from the study. In the SAkuraSky study, out of 83 NMOSD patients aged 12-74 years, 41 were administered Satralizumab 120 mg along with baseline immunosuppressant treatment (oral corticosteroids, azathioprine, or mycophenolate mofetil) and the rest 42 patients received matched placebo. The SAkuraStar study was conducted on 95 patients aged 18-74; 63 were allocated to the Satralizumab 120 mg monotherapy arm, and 32 were allocated to the placebo arm. In both studies, satralizumab or a matched placebo was administered subcutaneously at two-week intervals up to the fourth week of treatment (0, 2, and 4 weeks) and then at every four-week interval. Satralizumab and placebo group had a median treatment duration of 107.4 weeks (range 2-224) and 32.5 weeks (range 0 to 180), respectively, in the SAkuraSky trial and 92.3 weeks (range 0 to 202) and 54.6 weeks (range 2 to 216) in SAkuraStar trial during the double-blind period. However, the median treatment duration of all the patients receiving Satralizumab in the double-blind and extension periods was 143.1 weeks (range 15 to 224) in the SAkuraSky trial and 95.9 weeks (range 5 to 206 ) in the SAkuraStar trial [38]. The primary efficacy outcome in both studies was the first protocol-defined relapse (PDR). The PDR was adjudicated by a clinical end-point committee, and members of the committee had no knowledge about the trial-group assignments. The secondary efficacy endpoints in both studies were mean change in pain visual analogs scale (VAS) score and functional assessment of chronic illness therapy-fatigue (FACIT-F) score from baseline to week 24. Some other important outcome measures were changes in mean expanded disability status scale (EDSS) score, annualized relapsed rate (ARR), Zarit Burden Interview score, 36-item Short Form Health Survey score, uroQol-5 Dimensions (EQ-5D) instrument score, modified Rankin Scale, and change in visual acuity based on Snellen chart from baseline to week 24 [39].

In SAkuraSky study, eight patients receiving add-on satralizumab and 18 patients taking placebo developed protocol-defined relapse (20% vs. 43%) (95% CI, 0.16 to 0.88; p= 0.02, hazard ratio- 0.38) during the double-blind period. Similarly, in the SAkuraStar trial, 19 patients belonging to the satralizumab group and 16 patients belonging to the placebo group experienced protocol-defined relapse (30% vs. 50%) (95% CI, 0.23 to 0.89; p= 0.018, hazard ratio- 0.45) during the double-blind period. The patient population in both clinical trials consists of AQP4-IgG seropositive and seronegative groups. 

Subgroup analysis of primary efficacy outcome data from the SAkuraSky study revealed that a larger percentage of AQP4-IgG-seropositive patients in the placebo group developed relapse (12 of 28 patients, 43%) than those in the satralizumab group (3 of 27 patients,11%) (HR, 0.21; 95% CI, 0.06 to 0.75). As far as AQP4-IgG-seronegative patients are concerned, out of 28 AQP4-IgG-seronegative patients, 14 each had received satralizumab and a placebo. However, five (36%) patients on satralizumab and six (43%) patients on placebo experienced relapse. The hazard ratio was 0.66 (95% CI, 0.20 to 2.24). Similarly, in the SAkuraStar trial, 64 patients were AQP4-IgG seropositive, and among them, satralizumab was administered to 41 patients, and 23 patients received a placebo. PDR was encountered in 9 (22%) patients in the satralizumab arm and 13 (57%) in the placebo arm. (HR, 0.26; 95% CI, 0.11 to 0.63). In the AQP4-IgG seronegative subgroup, 10 (46%) of 22 patients receiving satralizumab had PDR compared to 3 (33%) of nine patients receiving placebo (hazard ratio, 1.19; 95% CI, 0.3 to 4.78) [35]. At 48 weeks, 89% (95% CI, 72.8 to 95.7) patients in the satralizumab group were free from relapse compared to 66% (95% CI, 47.7 to 79.3) in the placebo group, and at 96 weeks, the proportion of relapse-free patients was 78% (95% CI, 58.1 to 88.8) in the satralizumab arm in contrast to 59% (95% CI, 39.9 to 73.4) in the placebo arm in SAkuraSky trial. Similarly, in the SAkuraStar trial, 76% (95% CI, 64 to 85) patients on Satralizumab did not have relapse compared to 62% (95% CI, 43 to 76) patients on placebo at 48 weeks. At 96 weeks, this was 72% (95% CI, 59 to 82) versus 51% (95% CI, 32 to 67). 

Both studies demonstrated that NMOSD patients on satralizumab had a significantly reduced risk of relapse relative to placebo in the AQP4-IgG seropositive group. Similarly, a greater number of patients with satralizumab were free from relapse compared to placebo at 48 and 96 weeks in AQP4-IgG seropositive NMOSD patients. The same cannot be claimed for AQP4-IgG seronegative patients due to inadequate evidence.

When data from both SAkuraSky and SAkuraStar studies were pooled together and analyzed, it was found to be in agreement with those of individual studies. The result of pooled analysis suggested a 58% reduction in PDR among patients of the Satralizumab group compared with the placebo group (HR, 0.42; 95% CI, 0.25 to 0.71). Among the AQP4-IgG seropositive group, the reduction in PDR among patients treated with satralizumab was 75% (HR, 0.25; 95% CI, 0.12 to 0.50). Similarly, a significant difference was observed in the relapse-free rate between satralizumab and placebo groups at 48 weeks (81% vs. 64%) and 96 weeks (74% vs. 55%). However, no conclusive benefit was observed in the AQP4-IgG seronegative group in the pooled analysis; a possible explanation is the small sample size and heterogeneity within the subgroup [32,39,40].

As far as key secondary endpoints are concerned, no significant difference was observed in the VAS score (p= 0.52, SAkuraSky and p=0.44, SAkuraStar) and FACIT-F score from baseline to week 24 between Satralizumab and placebo groups [34,35]. The key characteristics of the clinical trials are depicted (Table 2).

**Table 2:** Characteristics of included studies Abbreviations: EDSS- Expanded Disability Status Scale; FACIT- Functional Assessment of Chronic Illness Therapy; NMO- Neuromyelitis Optica, NMOSD-Neuromyelitis Optica Spectrum Disorder; PDR- Protocol Defined Relapse; VAS- Visual Analogue Scale

Trial name	Reference	Phase	Age	Disease Population	Study design	Intervention	Comparator	Sample size	Key outcomes
SAkuraSky [34]	Yamamura et al. 2019 [34]	Phase III	Child Adult Older Adult: (12-74 years)	NMO NMOSD	Randomized, Double-Blind, Placebo-Controlled	Satralizumab at 0, 2^nd^, and 4^th^ week, and thereafter once every 4 weeks baseline Immunosuppressant	Placebo	83	PDR; VAS; FACIT; EDSS
SAkuraStar [35]	Traboulsee et al. 2020 [35]	Phase III	Adult: (18-74 years)	NMO NMOSD	Randomized, Double-Blind, Placebo-Controlled	Satralizumab at 0, 2^nd^, and 4^th^ week, and thereafter once every 4 weeks	Placebo	95	PDR; VAS; FACIT; EDSS

Safety

Safety and tolerability of satralizumab following 120 mg subcutaneous administration were observed and compared with that of matched placebo in both pioneer clinical studies. The key safety outcomes for both studies were adverse events, infections, injection-related reactions, and anaphylactic reactions. Relapse of disease was not classified as an adverse event. Satralizumab was found to be tolerated well in NMOSD patients with and without concomitant immunosuppressant. Both clinical trials established that the occurrence of adverse events in satralizumab and placebo groups was commensurate with each other.

The number of adverse events recorded per 100 patient-years (PY) in the satralizumab and placebo group was 485.2 and 514.3, respectively, in the SAkuraSky study. Similarly, a relatively higher proportion of serious adverse events was recorded in patients on placebo compared to patients on satralizumab treatment (20.2 events per 100 patients-years vs. 11.5 events per 100 patients-years). The incidence of adverse events recorded was 95% for the placebo arm and 90% for the satralizumab arm, and the incidence of serious adverse events was 21% and 17%, respectively, for the placebo and satralizumab groups. However, in contrast to the SAkuraSky study, relatively more adverse events and serious adverse events were reported in the satralizumab group than in the placebo group (473.3 vs. 495.2 events per 100 patient-years and 32.1 vs. 9.9 events per 100 patient-years) in the SAkuraStar study. The incidence of adverse events and serious adverse events recorded were (75% vs. 92% and 16% vs. 19%) for the placebo and satralizumab groups. 

When adverse events of both studies were pooled together and analyzed, it was found to be 478.49 events/100PY for Satralizumab and 506.51 events/100PY for placebo [34,35]. In both studies' urinary tract infection (UTI), upper respiratory tract infection (URTI), and infusion-related reaction (IRR) were the most common adverse events in either group. The incidence of all these adverse events was found to be more in patients receiving a placebo than in patients receiving satralizumab in the SAkuraStar trial, whereas it was contrary in the SAkuraSky trial. Neither any death nor any anaphylactic reactions were reported in either group during the double-blind period in both studies. Most of the IRRs were mostly mild-to-moderate and did not warrant discontinuation of treatment. Flushing, erythema, and pruritus were commonly reported IRRs [41]. Nasopharyngitis and headache are some of the other notable adverse events reported [34,35]. The pooled data indicated that 4 (6.8%) patients treated with Satralizumab and six (8.1) patients treated with placebo discontinued the studies on account of adverse events [39]. The details of adverse events of both studies are summarised (Table 3). 

**Table 3:** Reported adverse events Abbreviations: IRR- Infusion Related Reaction; UTI – Urinary Tract Infection; URTI- Upper Respiratory Tract Infection

	SAkuraSky trial [34]		SAkuraStar trial [35]	
Adverse events	Satralizumab (N=41)	Placebo (N=42)	Satralizumab (N=63)	Placebo (N=32)
UTI	7 (17.1%)	7 (16.7%)	11 (17.5%)	8 (25%)
URTI	10 (24.4%)	6 (14.3%)	10 (15.9%)	6 (18.8%)
Nasopharyngitis	10 (24.4%)	7 (16.7%)	9 (14.3%)	1 (3.1%)
Headache	10 (24.4%)	4 (9.5%)	10 (15.9%)	4 (12.5%)
IRR	5 (12.2%)	2 (4.8%)	8 (12.7%)	5 (15.6%)

## Conclusions

Monoclonal antibodies like eculizumab, inebilizumab, and satralizumab targeting complement C5, CD19, and IL-6, respectively, are emerging as promising therapeutic alternatives for managing NMOSD. It is evident from both clinical studies that satralizumab significantly reduced relapse rate in NMOSD patients, and this benefit is more marked in the AQP4-IgG-seropositive group. Furthermore, it is found to be safe and well-tolerated. The ease of administration and less frequent dosing schedule make it a promising drug for NMOSD patients.

However, satralizumab did not show a promising effect in AQP4-IgG-seronegative NMOSD patients compared to placebo. Moreover, both included studies had a modest number of participants without comparison with an active comparator. Hence, future trials addressing these concerns and evidence from real-world use will shed more light on the usefulness of this drug in NMOSD patients.
